# The Hepatic Transcriptomes of Two Mouse Models of Liver Fibrosis Reveal Shared Molecular Patterns Associated with a Common Dysregulation of Folate Metabolism

**DOI:** 10.1016/j.tjnut.2025.101349

**Published:** 2026-01-08

**Authors:** Robin P da Silva, Brandon J Eudy

**Affiliations:** 1 Department of Physiology and Pathophysiology, Rady Faculty of Health Sciences, University of Manitoba, Canada; 2 Previously affiliated with the Department of Food Science and Human Nutrition, University of Florida, Gainesville, FL, United States

**Keywords:** fibrosis, immune function, methionine, amino acids, one-carbon metabolism

## Abstract

**Background:**

Dysregulated one-carbon metabolism occurs in metabolic dysfunction-associated steatotic liver disease (MASLD) and in models of liver fibrosis, but two fibrosis models display opposing methylation cycle profiles, which has been a point of confusion. Broader changes in one-carbon related metabolism and the consequent impact on transcriptional events have not been fully explored.

**Objective:**

The objective of this study was to identify common metabolic and transcriptional profiles in methionine and choline deficient (MCD) and glycine N-methyltransferase knockout (GNMTKO) mice to help us understand molecular mechanisms that contribute to hepatic fibrosis.

**Methods:**

Eight-wk-old male GNMTKO (C57BL6J background) and control mice were fed AIN-76 based diet (24% casein, 60% sucrose/starch, and 5% fat) for 8 wk (*n* = 5–6). Ten-wk-old male C57BL6J mice were fed amino acid-defined diet (based on AIN-76) with or without sufficient methionine and choline (65% sucrose/starch, 15% defined amino acid, and 10% fat) for 5 wk (*n* = 6). We characterized the expression of folate metabolic enzymes, measured the amino acid content in plasma and liver, performed targeted metabolomics and RNA sequencing in liver to compare metabolite and transcriptional profiles.

**Results:**

We measured an 11-fold increase (*P* = 0.0067) in MTHFD1L1 and 2.8-fold (*P* = 0.013) MTHFS expression in liver of GNMTKO mice, matching results from our previous study in MCD mice. Liver mitochondria from GNMTKO mice had a 2-fold (*P* = 0.0423) higher capacity for oxidation of one-carbon units. We find common regulation of xenobiotic/metabolic sensors, growth, immune, and inflammatory pathways in our transcriptomic analysis. Statistical analysis was performed using an unpaired Student’s t-test with Welch’s correction, and RNA sequencing data were analyzed using the method of Benjamini-Hochberg.

**Conclusions:**

We identify a common dysregulation in folate metabolism in two widely used rodent models of liver fibrosis and highlight the consequent metabolic disturbances. Analysis of hepatic transcriptional profiles of MCD and GNMTKO mice reveals novel association of the transcriptional regulators STAT5b, AhR, and ARNT with liver fibrosis.

## Graphical abstract

**Figure:**
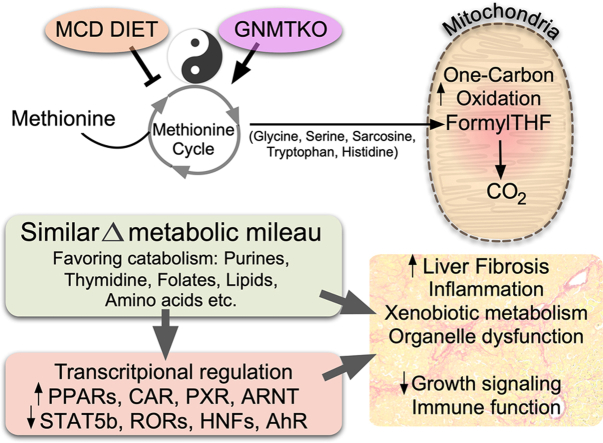

## Introduction

Metabolic dysfunction is strongly associated with fatty liver disease and liver fibrosis. Older disease diagnostic criteria have been refined, and the term nonalcoholic fatty liver disease (NAFLD) has been replaced with the more appropriate term: metabolic dysfunction-associated steatotic liver disease (MASLD). MASLD can progress to metabolic dysfunction-associated steatohepatitis (MASH), which is characterized by inflammation and accumulation of extracellular matrix (ECM/fibrosis), and both conditions are growing in prevalence [1]. Obesity and metabolic syndrome are strongly associated with MASH [2], but unfortunately, the underlying mechanisms that drive progression of this disease are not completely understood. Fibrosis is a major manifestation in MASH, and the extent of fibrosis is the best predictor of outcome, but there are few reliable and effective treatments for this condition. Weight loss in obese individuals with MASH has shown promise [3], and the novel glucagon-like peptide-1 (GLP-1) agonist antiobesity drugs are currently in clinical trials to treat MASH and cirrhosis [4]. Nonetheless, we must enhance our understanding of how metabolic dysregulation contributes to development of fibrosis if we hope to develop new therapies and treatments in the future.

Dysfunction of one-carbon (methionine and folate-mediated) metabolism is strongly associated with liver fibrosis [5]. Folate and B12 are essential cofactors for one-carbon metabolism, and having low status of these vitamins is highly correlated with histologically confirmed fibrosis in MASH [6]. Folate-dependent metabolism and methylation are also dysregulated in obesity, where MASLD and fibrosis have the highest prevalence, but how dysregulation of folate metabolism or methylation influences fibrosis is not entirely clear [2,7,8]. Research on liver disease has focused on impaired methylation and lipotoxicity as mechanisms contributing to fibrosis, but liver fibrosis is also observed in conditions where methylation is elevated. The methionine and choline deficient (MCD) diet and the knockout of glycine N-methyltransferase (GNMTKO) models have opposing hepatic methylation profiles, but both have significant liver fibrosis [5]. MCD mice have low hepatic content of S-adenosylmethionine (AdoMet), whereas GNMTKO mice have >20-fold increase in hepatic AdoMet content, indicating that methylation potential alone is likely not a core driver of fibrosis [5,9]. Our recent work using these models shows that MCD mice have a broad dysregulation in folate-dependent metabolism, and GNMTKO mice have indications of dysregulated folate-dependent metabolism that contribute to inflammation and fibrosis [5,10,11]. The goal of the current work was to establish the common metabolic changes in GNMTKO and MCD mice to give us insight into potential mechanisms that explain their similar hepatic phenotype. We profile hepatic metabolites and folate-dependent one-carbon metabolic enzymes, finding similarity in serine, glycine, purine, pyrimidine, and folate metabolism. Finally, we use a comparative approach to analyze RNA sequencing data from liver, revealing known and novel transcriptional pathways that are similarly regulated in these fibrosis models.

## Methods

### Animals

Male C57BL/6J wild-type (WT) and GNMTKO mice were weaned at 21 d, housed in ventilated cages with corn cob bedding, and maintained on a 10:14-h light/dark cycle. Before the experimental diets were introduced, mice were maintained on rodent diet (unpurified) (Teklad #2918) ad libitum until they reached ∼10 wk of age. All experimental diets were administered as outlined below, and detailed diet compositions are shown in Supplemental Table 1. Before tissue collection, all mice were deprived of food for 16 h and anesthetized with ketamine/xylazine.

GNMTKO mice: 8-wk-old WT and GNMTKO mice were provided ad libitum access to experimental diets for 8 wks. Diets were made using Envigo basal diet (without 20% oil by weight) mix (#TD88232 based on American Institute of Nutrition 76 (AIN-76); 24% casein, 30% sucrose, 15% corn starch, and 5% cellulose). The following additions were made to make the control diet consist of 1% lard, 1% canola oil, 3% corn oil, and 15% corn starch by weight as previously published [11]. This diet was designed to contain the same fat percentage as the maintenance (unpurified) diet but to otherwise have a similar ingredient profile to the AIN-76 diet. A separate group of GNMTKO (*n* = 3) and control (*n* = 3) mice, consuming Prolab RMH 3000 (unpurified, 22.5% protein, 32% carbohydrate, and 5.5% fat) ad libitum, were used for the mitochondrial respiration experiments.

MCD mice: at 10 wk of age, WT mice were randomly divided into two groups. One group was fed an amino acid-defined MCD diet (Envigo #TD.90262; based on AIN-76; 45% sucrose, 20% corn starch, 15% defined amino acid, 10% corn oil, and 3% cellulose). The control group was fed a matched control diet with the same defined amino acid content except with sufficient methionine and choline (Envigo #TD.94149) as previously published [10]. The MCD diet had a defined amino acid profile in place of casein to allow complete elimination of methionine and was chosen to maintain consistency with the broad research application of the MCD diet. Mice were fed the diets ad libitum for 5 wk. All study protocols were approved by the Institutional Animal Care and Use Committee of the University of Florida or the University of Manitoba and were in accordance with the Guide for the Care and Use of Laboratory Animals.

### RNA sequencing

Liver tissue was snap-frozen in liquid nitrogen prior to analysis. RNA was extracted using an RNEasy Plus Mini kit from Qiagen Inc. RNA quantity and quality were assessed using a Nanodrop One (Thermo-Fisher). Purified RNA samples were sent to Centre d’expertise et de services Genome Quebec (Montreal, QC) for bioanalysis and sequencing using an Illumina NovaSeq 6000 to a depth of 50 million reads. Sequences were aligned and analyzed using R studio and open-source code. Statistical analysis of data was performed with the assistance of the Bioinformatics core facility at the Children’s Hospital Research Institute of Manitoba. Pathway analysis of sequence data was performed using Ingenuity Pathway Analysis (IPA, Qiagen Inc.). Experimental groups were as follows: control WT (*n* = 6) compared with MCD (*n* = 6) or control WT mice (*n* = 6) compared with GNMTKO (*n* = 5); experiments were conducted independently, with a separate set of control mice used for each respective mouse model feeding experiment.

### Amino acid analysis

Amino acids were quantified as previously described [12]. Briefly, samples were treated with 6-aminoquinolyl-N-hydroxysuccinimidylcarbamate to make derivatives that were separated using an H-Class UPLC (Waters Inc.) equipped with an AccQ-Tag Ultra C18 Column, 1.7 μm, 2.1 × 150 mm column (Waters Inc.) and detected using a fluorescence detector. Norleucine was used as an internal standard and peaks were verified using individual purified amino acids.

### Metabolomics

Snap-frozen liver tissue was ground in liquid nitrogen and sent to the Southeastern Center for Integrated Metabolomics (SECIM) for targeted metabolomic analysis of nicotinic acid, purine, and pyrimidine nucleotides. All tissue treatments were performed according to SECIM protocols, where metabolites were separated by UPLC and quantified using single reaction monitoring compared against purified standards.

### Western blotting

Mouse livers were homogenized on ice, in cold radioimmunoprecipitation assay buffer containing protease and phosphatase inhibitors (Pierce Thermo-Fisher). Lysates were then diluted in Laemmli buffer containing 5% β-mercaptoethanol and heated at 95°C for 5 minutes. Protein content was measured using Bicinchoninic acid assay (Pierce Thermo Fisher). Proteins were resolved by SDS-PAGE, transferred to 0.45 um nitrocellulose membranes, and blocked in Tris-buffered saline with 0.1% Tween 20 (Biorad) with either 5% BSA or 5% skim milk powder. Blots were then incubated with primary antibodies overnight before being probed with an appropriate secondary antibody for 1 h. All antibodies were obtained from Cell Signaling Technologies except for those used to detect α-tubulin, serine hydroxymethyltransferases (SHMT) and methenyltetrahydrofolate synthease (MTHFS), which were obtained from Thermo Fisher Inc. Catalog numbers are as follows: α-tubulin (#A11126), Methylenetetrahydrofolate dehydrogenase 1 like (MTHFD1L) (#14999), Methylenetetrahydrofolate dehydrogenase 2 (MTHFD2) (#41377), MTHFS (#HPA008067), SHMT1 (#A304-259A), SHMT2 (#PA5-54232), thymidylate synthase (TYMS) (#9045), glycine decarboxylase (GLDC) (#12794), and protein disulfide isomerase (PDI) (#2446). The Clarity electrochemiluminescent substrate kit and Chemidoc MP imaging system (BioRad) were used for acquisition. Blot bands and density were analyzed using ImageLab software (BioRad).

### Mitochondrial respiration

Mitochondria from GNMTKO mice were isolated according to a previously described method [13]. Mitochondrial protein content was determined using the Bicinchoninic acid assay (Pierce Thermo-Fisher), and 0.8 mg mitochondrial protein was used for each experimental measurement. Either B-OH butyrate or glycine was added to mitochondria in respiration media to final concentration of 2.5 mM. ADP was added to a final concentration of 0.5 mM. Oxygen consumption was measured using an O2K respirometer and software (Oroboros Instruments).

### Statistical analysis

Statistics were performed using GraphPad Prism for all measurements, except for analysis of RNA sequencing data. RNA sequencing data were analyzed in R using DESeq2 and open-source online software, and the Benjamini-Hochberg method was applied. Otherwise, data are analyzed using unpaired Student’s t-test using Welch’s test. Data are expressed as mean ± SD unless otherwise indicated. *P* value of <0.05 indicates significance.

## Results

### Glycine and serine metabolism and broader folate-related metabolic enzyme expression are similarly dysregulated in GNMTKO and MCD mouse livers

**FIGURE 1:**
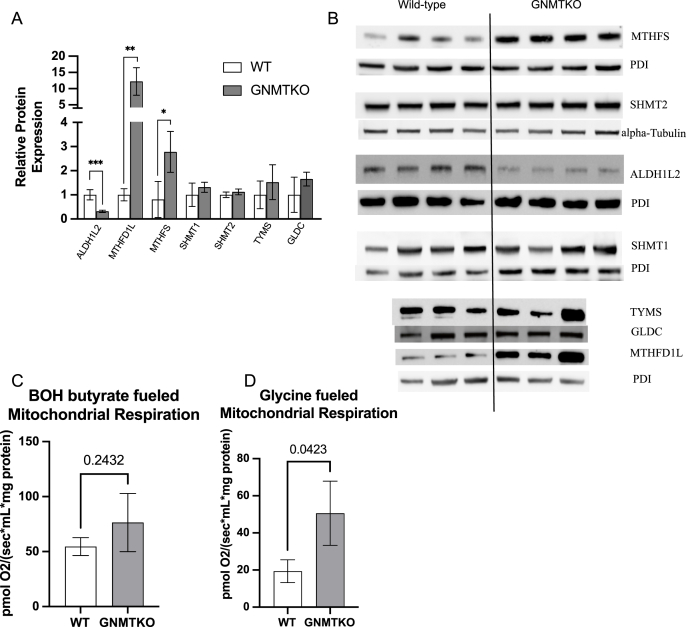
Expression of folate-related one-carbon metabolic enzymes in GNMTKO livers. (A), Quantification of integrated density values from Western blots. (B), Western blot for protein expression in liver. (C) mitochondrial respiration rates fueled by β-hydroxybutyrate and (D), glycine. Blots are normalized to loading control PDI. Values are presented as mean ± SD. Asterisks indicate significant difference (∗<0.05, ∗∗<0.01, ∗∗∗<0.001). ALDH1L2, Alcohol dehydrogenase 1L2; GLDC, glycine dehydrogenase; MTHFD1L, methylenetetrahydrofolate dehydrogenase 1L; MTHFS, methylenetetrahydrofolate synthetase; PDI, protein disulfide isomerase; SHMT1 or SHMT2, serine hydroxymethyltransferase; TYMS, thymidylate synthase; MCD, methionine and choline deficient; GNMTKO, glycine N-methyltransferase knockout.

In 2020, we reported a significant elevation in hepatic MTHFD1L1 and MTHFS enzyme expression in MCD mice and that additional disruption of folate metabolism in MCD-fed mice worsened liver fibrosis [10]. Using liver tissues from another 2020 study, we now show that GNMTKO mouse liver has a similar expression of MTHFD1L1 and MTHFS enzymes as was observed in liver from MCD mice (Figure 1A and B) [10,11]. We measured no significant changes in thymidylate synthase (TYMS), SHMT, or the glycine cleavage dehydrogenase (GLDC) in liver from GNMTKO mice; we previously found that TYMS was elevated and SHMT and GLDC were modestly decreased in MCD mice [10]. GNMTKO livers also had decreased expression of ALDH1L2 (not measured in MCD livers). Notwithstanding the small decrease in ALDH1L2 expression, oxidation of one-carbon units was higher in GNMTKO mice. Rates of βOH-butyrate oxidation in isolated liver mitochondria were not statistically different between control and GNMTKO mice (Figure 1C), but the capacity for oxidation of glycine was approximately double in GNMTKO compared with control liver mitochondria (Figure 1D), suggesting that there is elevated oxidation of one-carbon units in these mice.

### Serine and glycine metabolism are commonly regulated in MCD and GNMTKO mice

Analysis of plasma amino acids showed that glycine and serine were elevated in both GNMTKO and MCD fibrosis models and were the only common changes that we observed (Table 1). Hepatic glycine, glutamine, and ornithine were elevated in MCD mice compared with WT controls (Table 2), whereas GNMTKO liver had elevated serine, methionine, glutamate, glutamine, asparagine, histidine, and lower tyrosine content. The relatively large differences between amino acid values observed between MCD and GNMTKO mice are due to the free amino acid content of the amino acid-defined diet and severe catabolic nature of the MCD diet noted in our previous studies [10,11]. Nevertheless, disturbance in hepatic glycine in MCD liver and serine in GNMTKO liver was noted.

**TABLE 1:** Plasma amino acid concentrations in MCD and GNMTKO mice and respective control mice

Amino acid acidacidacid∖Group	Control^2^	MCD	P value	Control^3^	GNMTKO	*P* value
Alanine	ND	ND	ND	217.9 ± 56.6	271.5 ± 58.1	0.1214
Glycine∗	63.2 ± 8.2	165 ± 27.7	<0.0001^1^	52.8 ± 9.5	67.5 ± 7.3	0.0106^1^
Serine∗	32.5 ± 4.1	62.9 ± 9.8	<0.0001^1^	104.8 ± 16.0	131.0 ± 18.3	0.0188^1^
Sarcosine	ND	ND	ND	128.0 ± 44.4	127.7 ± 52.9	0.9927
Methionine	12.1 ± 1.9	23.9 ± 4.5	0.0001^1^	59.6 ± 25.1	63.8 ± 22.4	0.7559
Arginine	147.6 ± 30.0	13.9 ± 2.0	<0.0001^1^	51.5 ± 13.1	44.1 ± 11.8	0.3099
Ornithine	8.9 ± 5.1	10.2 ± 7.4	0.7397	18.5 ± 15.5	32.2 ± 13.6	0.1228
Citrulline	ND	ND	ND	1.7 ± 1.4	1.5 ± 1.7	0.8169
Asparagine	ND	ND	ND	60.4 ± 11.1	71.7 ± 9.6	0.0778
Glutamate	9.4 ± 2.1	9.4 ± 4.4	0.9778	20.1 ± 2.8	21.8 ± 5.7	0.4802
Glutamine	116.0 ± 15.4	155.5 ± 29.8	0.0165^1^	266.8 ± 27.5	287.1 ± 18.03	0.1503
Histidine	116.7 ± 15.6	156.2 ± 30.1	0.0171^1^	228.0 ± 101.5	197.9 ± 34.4	0.5054
Isoleucine	29.5 ± 5.5	40.9 ± 12.1	0.0621	64.6 ± 13.6	75.5 ± 19.9	0.2674
Leucine	41.6 ± 7.8	60.3 ± 19.5	0.0543	72.4 ± 14.8	86.7 ± 23.1	0.2024
Valine	57.5 ± 9.2	74.4 ± 21.1	0.1013	155.9 ± 23.3	172.1 ± 34.3	0.3334
Lysine	70.3 ± 9.4	110.8 ± 19.1	0.0009^1^	138.6 ± 21.8	159.6 ± 16.0	0.0772
Tyrosine	17.6 ± 2.5	22.9 ± 4.3	0.0473^1^8	45.7 ± 11.7	35.3 ± 13.0	0.1574
Phenylalanine	24.6 ± 2.1	28.4 ± 6.1	0.1765	61.0 ± 9.2	63.1 ± 5.6	0.6356
Proline	19.6 ± 4.0	34.8 ± 3.8	<0.0001^1^	53.3 ± 8.6	59.2 ± 6.3	0.1929
Hydroxyproline	ND	ND	ND	7.3 ± 2.2	10.2 ± 2.9	0.0691
Threonine	32.5 ± 27.5	119.3 ± 11.2	<0.0001^1^	41.0 ± 5.4	36.5 ± 2.6	0.0914
Amino adipic acid	ND	ND	ND	13.8 ± 5.6	12.6 ± 7.7	0.7413
Beta alanine	7.9 ± 3.1	33.9 ± 25.2	0.0494^1^	22.6 ± 4.7	30.7 ± 11.3	0.1095

Concentration presented as mean ± standard deviation in μM.

Abbreviations: GNMTKO, glycine N-methyltransferase Knockout; MCD, methionine and choline deficient diet fed mice; ND, not detected.

∗ An asterisk indicates the significance and direction of change in amino acid content are common to both mouse models.

1 *P* < 0.05 is considered significant, n = 6 per group.

2 Control matched diet control mice for MCD.

3 Control matched diet control mice for GNMTKO mice.

**TABLE 2:** Hepatic amino acid content in MCD and GNMTKO mice and respective control mice

Amino acid	Control^2^	MCD	P value	Control^3^	GNMTKO	*P* value
Alanine	2547 ± 863	2511 ± 1626	0.9566	89.5 ± 48.5	94.0 ± 30.5	0.8666
Glycine	241.5 ± 113.8	403.6 ± 169.5	0.0464^1^	562.0 ± 46.9	516.2 ± 115.5	0.4346
Serine	799.5 ± 253.1	932.9 ± 252.9	0.3635	207.5 ± 24.9	271.0 ± 21.6	0.0026^1^
Methionine	57.9 ± 19.8	77.4 ± 33.1	0.1840	35.6 ± 5.6	52.4 ± 11.3	0.0176^1^
Arginine	ND	ND	ND	ND	ND	ND
Ornithine	296.2 ± 57.4	417.1 ± 82.5	0.0146^1^	ND	ND	ND
Asparagine	398.1 ± 243.5	296.8 ± 214.3	0.5046	177.9 ± 18.1	129.9 ± 22.6	0.006^1^
Glutamate	1724 ± 574	2275 ± 1083	0.9778	452.1 ± 131.1	766.5 ± 66.7	0.0014^1^
**Glutamine**∗	4450 ± 1266	7242 ± 2998	0.0315^1^	2781 ± 166	4933 ± 1372	0.0072^1^
Histidine	3172 ± 1192	4025 ± 763	0.1291	228.0 ± 19.9	284.8 ± 38.9	0.0196^1^
Isoleucine	443.1 ± 264.2	500.6 ± 197.0	0.6902	38.2 ± 13.7	47.0 ± 16.2	0.3642
Leucine	488.8 ± 346.3	509.3 ± 248.3	0.9062	43.5 ± 10.4	51.8 ± 12.6	0.2868
Valine	418.5 ± 94.2	373.1 ± 161.2	0.5102	90.9 ± 20.1	113.1 ± 38.6	0.2873
Lysine	577.6 ± 149.2	801.8 ± 269.9	0.0635	155.0 ± 31.1	170.6 ± 27.8	0.4249
Tyrosine	149.9 ± 78.1	177.2 ± 68.1	0.4863	24.9 ± 5.6	13.9 ± 2.2	0.0037^1^
Phenylalanine	86.7 ± 18.4	113.7 ± 49.7	0.2395	35.0 ± 3.2	32.9 ± 4.1	0.3901
Proline	195.4 ± 52.39	222.2 ± 60.5	0.3728	33.8 ± 6.2	32.0 ± 2.7	0.5776
Hydroxyproline	ND	ND	ND	0.5 ± 0.2	1.0 ± 1.6	0.5027
Threonine	331.2 ± 175.7	359.0 ± 71.5	0.7748	85.7 ± 19.2	75.7 ± 26.7	0.5133
Beta alanine	ND	ND	ND	38.6 ± 21.8	76.2 ± 43.3	0.1304

Concentration presented as mean ± standard deviation in nmol/mg tissue.

Abbreviations: GNMTKO, glycine N-methyltransferase Knockout; MCD, methionine and choline deficient diet fed mice; ND, not detected.

∗ An asterisk indicates the significance and direction of change in amino acid content are common to both mouse models.

1 *P* < 0.05 is considered significant, n = 6 per group.

2 Control matched diet control mice for MCD.

3 Control matched diet control mice for GNMTKO mice.

### Nicotinamide, purine, and pyrimidine nucleotides in MCD and GNMTKO mouse livers

De novo purine and thymidine synthesis are dependent on one-carbon units from folate-dependent reactions. Our previously published global metabolomics pathway analysis in GNMTKO mice showed that purine metabolism was the most significantly altered pathway in liver [11]. Given that one-carbon units are integral components in nucleotide biosynthesis, we also sought to target these metabolites.

**FIGURE 2:**
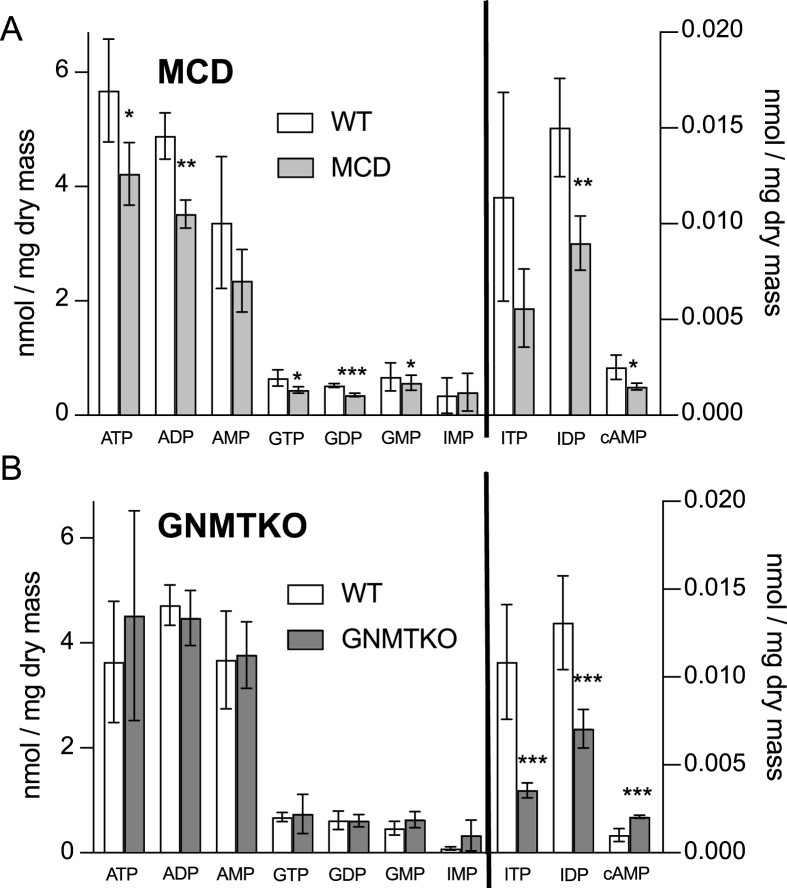
Targeted metabolomics of hepatic purine nucleotides from MCD and GNMTKO mice. Purine metabolites in liver expressed as nmole per mg dry liver tissue. White bars represent wild-type (WT) mice; Gray bars represent either MCD mice (panel A) or glycine-N-methyltransferase knockout (GNMTKO) mice (panel B). Data are presented as mean ± SD. Asterisks represents significant difference (∗< 0.05, ∗∗<0.01, ∗∗∗<0.001, *n* = 4 for MCD, and *n* = 6 for GNMTKO). ATP, adenosine 5’-triphosphate; ADP, adenosine 5’-diphosphate; AMP, adenosine 5’-monophosphate; cAMP, cyclic AMP; GTP, guanosine 5’-triphosphate; GDP, guanosine 5’-diphosphate; GMP, guanosine 5’-monophosphate; ITP, Inosine triphosphate; IDP, inosine diphosphate; IMP, inosine monophosphate; MCD, methionine and choline deficient; GNMTKO, glycine N-methyltransferase knockout.

**FIGURE 3:**
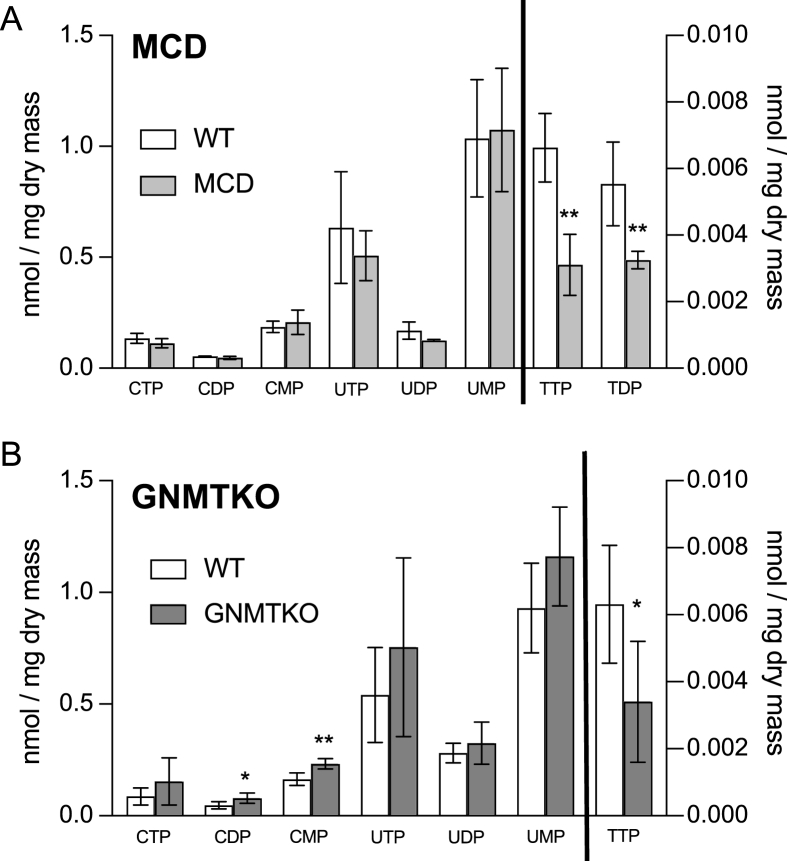
Targeted metabolomics of hepatic pyrimidine nucleotides from MCD and GNMTKO mice. Pyrimidine metabolites in liver expressed as nmole per mg dry liver tissue. White bars represent wild-type (WT) mice; gray bars represent either MCD mice (panel A) or glycine-N-methyltransferase knockout (GNMT^KO^) mice (panel B). Data are presented as mean ± SD. Asterisks represents significant difference (∗<0.05, ∗∗<0.01, ∗∗∗<0.001, *n* = 4 for MCD and *n* = 6 for GNMTKO). CTP, cytidine 5’-triphosphate; CDP, cytidine 5’-diiphosphate; CMP, cytidine 5’-monophosphate; TTP, thymidine 5’-triphosphate; TDP, thymidine 5’-diphosphate; TMP, thymidine 5’-monophosphate; UTP, uridine 5’-triphosphate; UDP, uridine 5’-diphosphate; UMP, uridine 5’-monophosphate; MCD, methionine and choline deficient; GNMTKO, glycine N-methyltransferase knockout.

The content of several purine nucleotides was decreased in MCD liver, including ATP, ADP, cyclic AMP (cAMP), GTP, GDP, guanosine 5’-monophosphate (GMP), and inositol 5’-diphosphate (IDP) (Figure 2A). In comparison, GNMTKO mice showed no significant changes in adenine or guanine nucleotide content but did show similar decreases in inosine 5’-triphosphate (ITP) and IDP (Figure 2B). With respect to pyrimidines, the only change shared between GNMTKO and MCD mice was a significant decrease in hepatic thymidine 5’-triphosphate (TTP) (Figure 3A and B). Thymidine 5’-diphosphate (TDP) content was also decreased in MCD liver. Modest increases in cytidine 5’-diphosphate (CDP) and cytidine 5’-monophosphate (CMP) were observed in GNMTKO livers. Interestingly, there was an elevation in cAMP in GNMTKO liver, but there was a decrease in cAMP in MCD liver. These differences in cAMP could be explained by lack of the substrate ATP in MCD mice. Overall, there is a decrease in inosine and thymidine nucleotide content common to both fibrosis models that may be the result of insufficient one-carbon units directed toward biosynthesis of thymidine and inosine nucleotides. However, other factors such as increased nutrient catabolism and overall decreases in anabolic reactions may also factor in decreased purine and pyrimidine biosynthesis.

**FIGURE 4:**
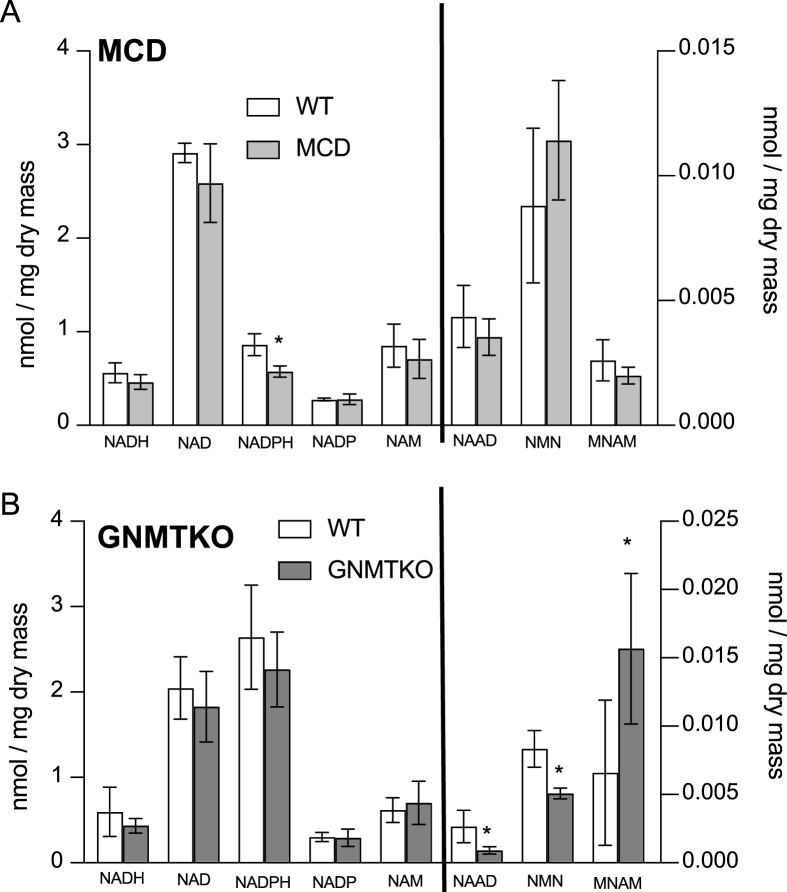
Targeted metabolomics of hepatic nicotinamide metabolites from MCD and GNMTKO mice. Metabolites in liver expressed as nmole per mg dry liver tissue. White bars represent wild-type (WT) mice; gray bars represent either MCD mice (panel A) or glycine-N-methyltransferase knockout (GNMTKO) mice (panel B). Data are presented as mean ± SD. Asterisks represents significant difference (∗<0.05, ∗∗<0.01, ∗∗∗<0.001, *n* = 4 for MCD and *n* = 6 for GNMTKO). NAD, nicotinamide adenine dinucleotide; NADH, nicotinamide adenine dinucleotide (reduced); NADP, nicotinamide adenine dinucleotide phosphate; NADPH, nicotinamide adenine dinucleotide phosphate (reduced); MNAM, 1-methylnicotinamide; NAAD, nicotinic acid adenine dinucleotide; NAM, nicotinamide; NMN, nicotinamide mononucleotide; MCD, methionine and choline deficient; GNMTKO; glycine N-methyltransferase knockout.

Given the known differences in methylation potential between these models and the role of methylation in nicotinamide degradation, it was not surprising that we did not find overlap in hepatic nicotinamide metabolites. Liver from MCD mice only showed a significant decrease in NADPH (Figure 4A), whereas liver from GNMTKO mice only displayed significant decreases in biosynthetic/salvage metabolites nicotinic acid adenine dinucleotide (NAAD) and nicotinamide mononucleotide, along with an increase in the concentration of the degradation product 1-methylnicotinamide. This confirms our previous finding, and the findings of others, that there is hypermethylation of nicotinamide in GNMTKO mouse liver [11,14]. Overall, there were no notable similarities in nicotinamide metabolites between the 2 models.

### Hepatic RNA sequencing in MCD mice

We identified 15,465 transcripts in liver samples from MCD mice and found that 2339 transcripts displayed significant differential expression. Pathway analysis in MCD mice identified canonical transcriptional pathways (Supplemental Table 2). MCD mouse liver displayed transcriptional patterns indicative of increased oxidative stress and inflammatory pathways, and decreases in transcripts associated with growth, proliferation, and immune function (Supplemental Table 3). Transcript patterns associated with disease and function were identified through IPA analysis, showing activation of angiogenesis, lipid synthesis, carbohydrate metabolism, tumor growth, and a decrease in organismal death (Supplemental Table 4).

### Hepatic RNA sequencing in GNMTKO mice

In GNMTKO liver we positively identified 15,477 transcripts with 844 transcripts displaying significant differential expression compared with control liver. The top 5 significant canonical pathways identified in GNMTKO livers are shown in Supplemental Table 5. GNMTKO mouse liver had elevated transcriptional patterns indicating increased succinylation, macronutrient catabolic reactions, and oncogenic pathways, and downregulation of transcripts associated with regulation of nutrient metabolism, liver growth/function, DNA metabolism, and immune function. Upstream canonical transcriptional regulators are shown in Supplemental Table 6. Disease and function analysis of GNMT mouse liver transcript revealed stimulation of pluripotency of stem cells and tumor growth with significance in noninsulin dependent diabetes, neurologic disease, ECM organization, lipid metabolism, and cell cycle function (Supplemental Table 7).

### Comparative analysis of RNA sequences between MCD and GNMTKO liver

Given that the MCD mouse model is a severe dietary deficiency and GNMTKO mouse model is a genetic metabolic model, we expected that they would have very different transcriptional profiles in the liver, but we thought that important information would be found in the common transcriptional changes. We found that compared with their controls, GNMTKO and MCD mice had a total of 188 common differentially expressed transcripts, with 126 similarly upregulated and 50 similarly downregulated (Supplemental Table 8). The upregulated transcripts are known to be involved in microtubule cytoskeletal organization, inflammatory cytokine production, nuclear factor κβ (NF-κβ) signaling, ER-associated degradation, protein glycosylation, and intracellular zinc transport. The downregulated transcripts were associated with lipid metabolism (retinol, arachidonic acid, and cholesterol), lysine acetyltransferase, glycosyl and glucuronosyltransferase, serine peptidase inhibitors, growth hormone (GH) signaling, IL signaling, NF-κβ signaling, nuclear factor erythroid 2-related factor (NRF2) regulation, angiogenesis, complement activation, histone methylation, T-cell survival, and transcriptional regulators controlling organ development, including hepatic duct development. Finally, several major urinary protein transcripts, equivalent to human lipocalins, known to influence metabolism and glucose homeostasis in mice, were downregulated in the liver [15]. There were 12 inversely regulated transcripts, of which 11 were upregulated in GNMTKO but downregulated in MCD liver. These opposing transcripts may represent transcripts that are influenced by differences in methylation reactions, given the opposite methylation profile observed between these models.

Finally, we conducted pathway analysis of common transcripts in MCD and GNMTKO livers. The top 5 common pathways were aryl hydrocarbon receptor (AhR) signaling, LPS/IL-1 mediated inhibition of retinoid X receptor (RXR) function, NRF2-mediated oxidative stress response, nicotine degradation II, glutathione-mediated detoxification (Supplemental Table 9). The extended list of common regulated pathways included serotonin, melatonin pathways of tryptophan metabolism, and pathways crucial for immune function, including acute phase response and the complement system.

**FIGURE 5:**
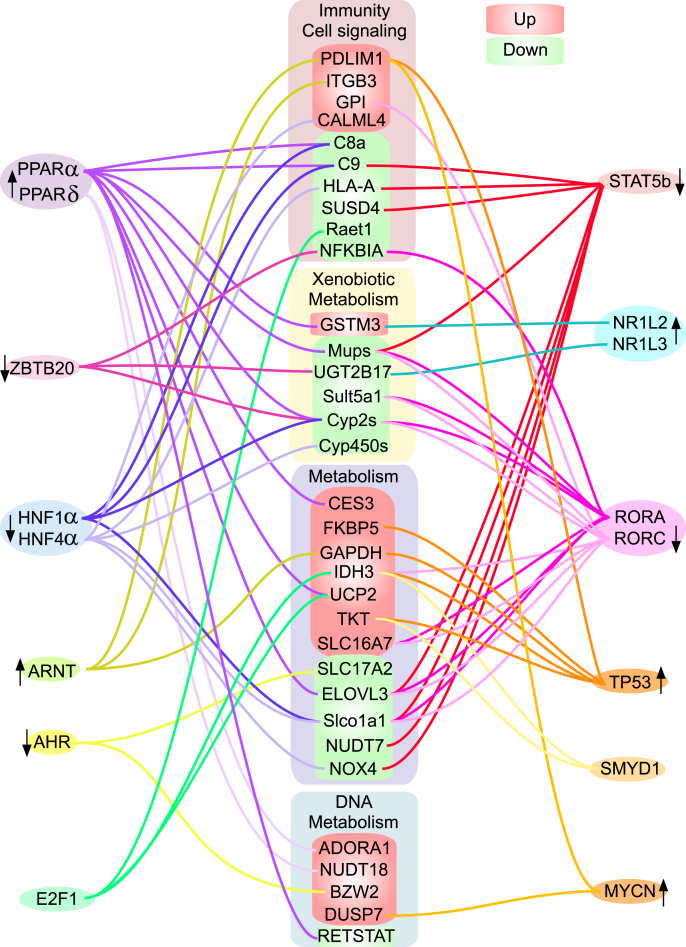
Summary of common transcriptional regulators identified in liver of MCD and GNMTKO mice. RNA sequence data from livers of GNMTKO and MCD mice were compared using Ingenuity Pathway Analysis software. Common transcripts are shown in the middle rectangular boxes (red indicates increased transcript and green indicates decrease in transcript). Transcription factors are shown in ovals around the outside and connected to common transcripts. Black arrows indicate the direction of transcriptional activity.

The upstream regulators that overlapped the fibrosis models are shown in Figure 5 and included signal transducer and activator of transcription 5b (STAT5b), peroxisome proliferator-activated receptors (PPARs), receptor tyrosine kinase-like orphan receptors (RORs), hepatic nuclear factors (HNFs), the AhR, and the aryl hydrocarbon receptor nuclear translocator. The full detailed list of common transcripts detected in MCD and GNMTKO mice is shown in Supplemental Table 10. PPARα had the most significant activation in fibrosis according to Z-score and increased activity of PPARδ was also observed. The orphan receptors nuclear receptor subfamily 1, group I, member 2 (a.k.a pregnane X receptor) NR1L2/PXR and nuclear receptor subfamily 1, group I, member 3 (a.k.a. constitutive androstane receptor) NR1L3/ constitutive androstane receptor (CAR) were activated in these models. N-myc proto oncogene (MYCN) and transformation-related protein (p53/TP53) were both increased, indicating response to DNA damage and cell cycle disruption. The strongest predicted decrease in activity was for STAT5b, suggesting insufficient GH signaling. RORA, RORC, HNF1α, and HNF4α activity appeared to be decreased, supporting the notion that liver development is impaired. Finally, the AhR and aryl hydrocarbon receptor nuclear translocator had significant overlap in MCD and GNMTKO mouse liver, with transcripts indicating decreased AhR activity and increased ARNT activity. This agreed with our previous metabolomic analysis, where we identified aberrant tryptophan metabolism in GNMTKO mice and found that canonical AhR transcriptional targets were decreased in the liver [11].

## Discussion

The influence of very high or very low AdoMet on liver damage was extensively reviewed by Lu et al. [9], and it was noted that the mechanisms connecting these phenomena were not clear. In a recent review, we postulated that changes in the broader folate-mediated one-carbon reactions may contribute to the fibrosis phenotype in GNMTKO and MCD models [5]. We also reported that enhancing disruption of folate metabolism in MCD mice worsened the fibrosis phenotype and that expression of one-carbon metabolic enzymes favored mitochondrial formate production in MCD mouse liver [10]. We now show that GNMTKO mouse liver expresses a profile of folate metabolic enzymes that is similar to MCD liver. GNMTKO mice have elevated hepatic expression of MTHFD1L and MTHFS, and a greater capacity for formate (one-carbon units from glycine) oxidation in liver mitochondria. Our amino acid data indicate that plasma glycine and serine are elevated, suggesting either decreased uptake of these amino acids for protein synthesis or increased protein catabolism in these fibrosis models. Together with our previous work, these data indicate that both GNMTKO and MCD mice favor mitochondrial formate production and oxidation despite opposing methylation potentials. It is not known whether a similar dysregulation of folate metabolism occurs as a result of mitochondrial dysfunction in human MASLD and MASH, but notable changes in folate metabolism occur in these conditions and in associated comorbidities. Serum folate is low in obesity (81%; CI: 55, 94) and type-2 diabetes (T2D) (54%; CI: 37, 70), where MASLD and MASH are prevalent [2,7,16]. It has also been demonstrated that folate supplementation mitigates liver inflammation and reduces liver fibrosis in rodent obesity models [17,18]. These findings suggest that dysfunctional folate metabolism is a potential contributing factor to the accumulation of fibrosis in liver disease.

Net loss of lean mass, or impaired growth, may be a consequence of the changes in folate metabolism that we observe. RNA sequencing analysis revealed transcriptional patterns affecting growth, insulin signaling, angiogenesis, and catabolism (Supplemental Tables 3 and 6). Our previous study showed that GNMTKO mice have reduced food efficiency and are resistant to weight gain on a high-fat diet, indicating that their metabolic rate is elevated and that they are catabolizing more macronutrients, including amino acids, compared with WT mice [11]. Hughey et al. [14,19] estimated that flux through gluconeogenesis and the Tricarboxylic acid (TCA) cycle was reduced in GNMTKO mice, but their technique used a short-duration fast (8 h) under sedation, so it was not possible to draw a definitive conclusion regarding net amino acid oxidation or energy balance across a 24 h period. Loss of total body mass from tissue catabolism, brought about by a deficiency of an essential amino acid, as is the case in MCD mice, must also be accompanied by increased hepatic amino acid catabolism [10,20]. Our collective data indicate that GNMTKO and MCD mice have elevated catabolic metabolism that either slows growth in the case of GNMTKO mice or causes net weight loss in the case of MCD mice.

Reduced nucleotide biosynthesis and DNA damage have been previously characterized in GNMTKO and MCD mice [20,21]. Increased oxidation of one-carbon units likely occurs at the expense of *de novo* purine and thymidine synthesis in these fibrosis models. MCD mice had disturbance in hepatic content of a larger number of nucleotide species compared with GNMTKO mice, most likely due to harsh nature of the MCD diet. However, the magnitude of change in similarly altered nucleotides was approximately the same. These findings are likely due to the nature of the homeostatic mechanisms controlling nucleotide metabolism within the tolerances necessary for life. A similar profile of transcripts that indicate increased activity of TP53 and MYCN, which are known responders to DNA damage, was found in both MCD and GNMTKO mouse livers. Further, we observe similar expression of transcripts influenced by the histone methyltransferase SET and MYND domain-containing 1 (SMYD1) that are also associated with DNA damage [22]. SMYD1 is regulated by SUMOylation events that may be altered by the dysregulated folate metabolism that we observe [10,[23], [24], [25]]. Our findings support the notion that a dysregulation of nucleotide biosynthesis resulting in DNA damage is a contributing factor to inflammation and oncogenesis that are observed in these mouse models.

Our analysis revealed several transcriptional pathways previously implicated in mechanisms of liver fibrosis (Figure 5). PPARα agonists like pioglitazone are used as therapies for MASLD, but unfortunately have limited efficacy [[26], [27], [28]]. PPAR molecules bind to retinoic acid receptors (RXRs), which are associated with stellate cell activation and fibrogenesis [29]. The PPARα activation in MCD mice that we observe may be a compensatory mechanism in response to metabolic stress in the liver, and perhaps differences in human PPAR activity are the reason why therapies targeting these pathways have had mixed effects on fibrosis.

RORA has been identified as an important molecule involved in differentiation of hepatic stellate cells into myofibroblasts [30]. RORA is negatively associated with liver fibrosis in humans, and low activity of RORA and RORC was found in a rodent model of fibrosis caused by a genetic defect in cholesterol synthesis [31]. Low RORC activity has also been observed in thioacetamide induced liver fibrosis [32]. Exploration into mechanisms that regulate ROR activity in fibrosis could be a path to novel therapies that deserve more attention.

HNFs are key transcriptional factors that drive changes in liver metabolism and development. Variants of HNF1α have been characterized and show metabolic dysregulation like that associated with impaired insulin signaling [33]. HNF4α is closely associated with MASLD and is found to be significantly depressed in liver fibrosis and cirrhosis [34]. Both HNF1α and HNF4α transcriptional targets are low in the MCD and GNMTKO liver. It is unclear whether HNFs may be useful therapeutic targets for fibrosis, but there is some evidence that they may protect against inflammation and lipotoxicity.

Elevated transcriptional targets of NR1L2, known as the pregnane X receptor (PXR), and another related family member NR1L3, known as CAR, were found in both fibrosis MCD and GNMTKO liver. PXR has been identified as an important factor in chronic liver disease implicated in liver regeneration, hepatocyte proliferation, and regulation of NF-κβ-associated inflammation [35]. CAR is known to drive xenobiotic and endobiotic clearance but is also known to play an important role in tissue regeneration [36]. CAR knockout mice fed a MCD diet did not develop significant fibrosis and had decreased plasma alanine aminotransferase (ALT) activity [37]. These findings are interesting because neither MCD nor GNMTKO mice were treated with xenobiotic compounds and yet display elevated PXR and CAR-associated transcriptional targets, suggesting that these transcription factors may be regulated by endogenous or microbial metabolites in liver. PXR and CAR are still regarded as orphan receptors with only a few sterol-related molecules identified as putative ligands, but none are known to bind under physiological conditions [35,38]. Like PPARs, PXR and CAR both dimerize with RXR [36], and these relations to retinoid metabolism may all work together to promote liver fibrosis. Changes in the metabolic milieu brought about by dysregulated folate metabolism could increase or decrease ligands for these orphan receptors or other transcription factors identified in our analysis, but intensive molecular characterization is required to establish their biological importance.

A few regulators with no previously known role in fibrosis were identified in our comparison. The largest and most significant decrease was observed for transcripts associated with STAT5b. STAT5b is known to be activated by the GH receptor and is known to regulate transcripts associated with immune cell proliferation, hematopoiesis, immune development, and Th17 differentiation [39]. We have previously found that circulating immune cells are decreased in GNMTKO mice and that MCD mice have reduced circulating cytokine concentrations [10,11]. Insufficient immune cell proliferation or immune response may contribute to accumulation of fibrosis because immune cells are responsible for providing pathogen defense, mediating tissue turnover, and breaking down ECM to make way for new cells. A deficit in GH signaling would allow for increased catabolism of amino acids, but we do not know what factors are causing a deficiency of GH signaling in MCD or GNMTKO mice. It is possible that altered folate metabolism may influence somatostatin or GH pathways, preventing adequate immune cell proliferation and liver growth/regeneration. Importantly, we observed deficits in transcripts for complement factors and signaling molecules, including those that affect the initial steps of the classical and lectin activation of the complement pathway and integral members of the downstream membrane attack complex that are crucial for innate pathogen defense. Deficient STAT5b and HNF activity appear to be contributing to a lack of complement factors (Figure 5) and may be synergistically responsible for these observations. It is possible that an insufficient immune response may be a more important factor in the development of fibrosis in these models than the amount of DNA damage-associated cell death.

We previously identified a dysregulation of AhR in GNMTKO mice [11]. AhR plays an important role in liver development [[40], [41], [42]], and our current transcriptional analysis indicates that AhR targets are significantly reduced in MCD and GNMTKO models. Somewhat counterintuitively, transcripts regulated by ARNT were elevated in MCD and GNMTKO mice. ARNT was initially characterized as binding to AhR during canonical signaling in response to environmental toxins established as hepatotoxic and inducing liver fibrosis [43,44]. However, ARNT was later found to have independent transcriptional activity and has been associated with increased expression of Integrin β-3 (ITGB3) [45], which we found upregulated in both fibrosis models (Figure 5). Integrins are key proteins expressed by hepatic stellate cells involved in regulating ECM formation [46] and have been proposed as putative drug targets for therapeutics to treat fibrosis [47]. Tryptophan metabolites are known ligands of AhR, and dysregulation of tryptophan metabolism has been identified in MASLD and MASH [48]. Tryptophan catabolism may be similarly affected in MCD and GNMTKO mouse liver, given the enhanced amino acid catabolism that occurs in weight loss (MCD mice) and slow growth (GNMTKO mice). It is possible that changes in tryptophan metabolism are negatively influencing AhR/ARNT signaling. Given that the AhR/ARNT axis has been implicated in mechanisms of fibrosis, the relationship between tryptophan metabolism and these signaling pathways represents an important area of research.

Despite decades of research, we do not have a complete understanding of the factors that influence the development of fibrosis. Even accurately staging liver fibrosis is still only possible through invasive tissue biopsy. This is a major problem given the high prevalence of MASLD and the fact that only 20% of individuals with MASLD progress to MASH [49,50]. Further, only ∼15% of individuals with MASH progress to end-stage liver disease, and a similar heterogeneity of progression to fibrosis is observed in viral associated hepatitis. Although differences in outcome are due in part to the duration of insult and the extent of damage, the capacity for tissue repair that is directed by the immune system must also play a major role. Metabolic dysregulation may have an influence on immune function through some of the pathways that we identified in this study. These pathways and molecules may be exploited to improve disease staging methods or be targeted to develop novel therapies.

In conclusion, this study greatly improves our understanding of two models of liver fibrosis, including the widely used MCD diet model. We now show that a similar dysregulation of mitochondrial folate metabolic enzymes occurs in MCD and GNMTKO mice. Comparative RNA sequencing analysis revealed common regulation of key transcription factors, including several canonical responses to xenobiotic/metabolic stimulus, now recognized to play important roles in liver and immune system. Although impaired nucleotide biosynthesis and DNA damage contribute to liver damage, there are broader metabolic consequences that we do not fully understand. The pathways and factors that we have identified may be influenced by dysregulated metabolism that together contribute to accumulation of fibrosis in the liver. Mitochondrial dysfunction and a dysregulation of folate metabolism are known to be present in MASLD, and the consequent changes in the metabolic milieu may drive some of the same transcriptional events that we observe in these mouse models of fibrosis [5,7,8,51]. Further research is necessary so that we can better understand how folate species and related metabolites might influence liver disease.

## Author contributions

The authors’ responsibilities were as follows – BJE: animal husbandry, tissue processing, Western blotting, HPLC analysis, writing methods text and provided comments on the manuscript; RDS: study design, surgical procedures, analysis of RNA sequencing, HPLC analysis, data analysis, figure composition, and writing of the manuscript. Both authors read and approved the final version of the manuscript.

## Data availability statement

Data are available on request from the corresponding author. Sequencing data are stored in the Borealis data repository.

## Funding

Funding was in part from the Rady Faculty of Health Sciences through Department of Physiology and Pathophysiology, the Manitoba Medical Services Foundation, and a National Science and Engineering Research Council (NSERC) Discovery grant. This work was also supported in part by seed funding from the University of Florida Institute of Food and Agricultural Sciences (IFAS), the IFAS Office of Research, and a pilot grant from the Southeast Center for Integrated Metabolomics (SECIM).

## Conflict of interest

RdS reports financial support was provided by Southeast Center for Integrated Metabolomics, Natural Sciences and Engineering Research Council of Canada, and Manitoba Medical Services Foundation. BJE has no conflict of interest to declare.
