# Supplementary material for: The Hepatic Transcriptomes of Two Mouse Models of Liver Fibrosis Reveal Shared Molecular Patterns Associated with a Common Dysregulation of Folate Metabolism
Source: J Nutr. 2026 Jan 8;156(3):101349. doi: 10.1016/j.tjnut.2025.101349 (PMC13014510; doi:10.1016/j.tjnut.2025.101349)
Supplement: Multimedia component 1 [file mmc1.docx]

**Title: Comparative transcriptomics from liver fibrosis models reveal molecular patterns associated with dysregulation of folate metabolism**

**First Author: Robin da Silva**

**Supplemental Table 1**. Experimental diet compositions

| Proximate values (as % Kcal) | Control diet^1^  (GNMTKO^2^) | Amino acid control diet | MCD diet |
| --- | --- | --- | --- |
| Protein | 25.2 | 15.4 | 14.9 |
| Carbohydrate | 63.0 | 63.1 | 63.2 |
| Fat | 11.8 | 22.1 | 22.1 |
| Kcal/g | 3.8 | 4.0 | 4.1 |
| Ingredient (g/kg diet) |  |  |  |
| Sucrose | 298.8 | 443.6 | 455.3 |
| Corn starch | 300 | 198.8 | 200 |
| Casein | 240 | 0 | 0 |
| Individual amino acid (without Methionine) | 0 | 135.4 | 135.4 |
| Vitamin Mix^3^ | 12.0 | 10 | 5.0 |
| Vitamin E (acetate) | 0 | 0 | 0.242 |
| Vitamin A (palmitate) | 0 | 0 | 0.04 |
| Vitamin D3 | 0 | 0 | 0.0044 |
| Mineral Mix (#170915) | 41.7 | 35 | 35 |
| Calcium Carbonate | 3.4 | 0 | 0 |
| Calcium Phosphate Dibasic | 0 | 3 | 3 |
| DL-Methionine | 3.6 | 8.2 | 0 |
| Cellulose | 50 | 30 | 30 |
| Canola Oil | 10.0 | 0 | 0 |
| Corn oil | 30.0 | 100 | 100.0 |
| Lard | 10.0 | 0 | 0 |
| Ethoxyquin, antioxidant | 0 | 0.02 | 0.02 |
| ^1^ Diet made from basal diet mix *without 20% oil TD.88232 from Harlan Teklad (Indianapolis, IN, USA). Fat sources and additional corn starch were purchased from Safeway Inc.  ^2^ Diets fed to GNMTKO mice and their respective C56BL6J controls  ^3^ Vitamin mix was #40060 for the basal diet mix and the amino acid control diet. A modified vitamin mix from Teklad #83171 without choline, vitamin A, D or E was used in the MCD diet.  Methionine and choline deficient; MCD, glycine N-methyltransferase knockout mouse; GNMTKO, Kilocalorie; Kcal | | | |
